# Supplementary material for: Supply-side barriers to maternal health care utilization at health sub-centers in India
Source: PeerJ. 2016 Nov 3;4:e2675. doi: 10.7717/peerj.2675 (PMC5101621; doi:10.7717/peerj.2675)
Supplement: Table S1 [file peerj-04-2675-s001.docx]

**Table A1: Variance inflation factors for antenatal care utilization model.**

| **Variables** | VIF | 1/VIF |
| --- | --- | --- |
| **Health personnel** |  |  |
| *Nurse Midwife* |  |  |
| *None* | 1.12 | 0.892 |
| *Only Contractual ANM* | 1.11 | 0.899 |
| *Both* | 1.13 | 0.888 |
|  |  |  |
| **Drug availability** |  |  |
| *Paracetamol* |  |  |
| *No* | 1.28 | 0.782 |
| *IFA* |  |  |
| *No* | 1.36 | 0.736 |
|  |  |  |
| **Equipment** |  |  |
| *BP instrument* |  |  |
| *Yes* | 1.4 | 0.712 |
| *Weighing scale* |  |  |
| *Yes* | 1.36 | 0.733 |
| *Examination table* |  |  |
| *Available but unusable* | 1.09 | 0.919 |
| *Not available* | 1.29 | 0.776 |
| *Bed Screen* |  |  |
| *Available but unusable* | 1.16 | 0.863 |
| *Not available* | 1.31 | 0.760 |
|  |  |  |
| **Infrastructure** |  |  |
| *Electricity* |  |  |
| *Irregular supply* | 1.92 | 0.521 |
| *No connection* | 2.28 | 0.437 |
| *Water supply* |  |  |
| *Yes* | 1.13 | 0.881 |
| *Toilet* |  |  |
| *Yes* | 1.22 | 0.817 |
| *Telephone* |  |  |
| *Yes* | 1.23 | 0.813 |
|  |  |  |
| ***Quality variables*** |  |  |
| *ISD training in last 5 years* |  |  |
| *Yes* | 1.12 | 0.891 |
| *VHSC monitoring work* |  |  |
| *Yes* | 1.23 | 0.810 |
|  |  |  |
| ***Other variables*** |  |  |
| *Region* |  |  |
| *Central* | 4.78 | 0.209 |
| *Northeast* | 1.76 | 0.567 |
| *East* | 2.56 | 0.390 |
| *West* | 2.08 | 0.480 |
| *South* | 2.69 | 0.372 |
| *ANM's residence from SC (in km.)* |  |  |
| *5-20* | 1.16 | 0.860 |
| *21-40* | 1.07 | 0.933 |
| *>40* | 1.02 | 0.978 |
|  |  |  |
| **Socioeconomic variables** |  |  |
| *Log of catchment population* | 1.20 | 0.835 |
| *% population in lowest wealth quintile* | 1.22 | 0.821 |
| *Total fertility rate* | 2.65 | 0.377 |
| *% Hindu population* | 1.56 | 0.642 |
| *Maternal education (in years)* | 2.45 | 0.407 |
| ***Mean VIF*** | **1.61** | |
