# Supplementary material for: Supply-side barriers to maternal health care utilization at health sub-centers in India
Source: PeerJ. 2016 Nov 3;4:e2675. doi: 10.7717/peerj.2675 (PMC5101621; doi:10.7717/peerj.2675)
Supplement: Table S2 [file peerj-04-2675-s002.docx]

**Table A2: Variance inflation factors for delivery care utilization model.**

| **Variables** | **VIF** | **1/VIF** |
| --- | --- | --- |
| **Health personnel** | |  |
| Nurse Midwife | |  |
| *None* | 1.10 | 0.910847 |
| *Only Contractual ANM* | 1.10 | 0.908165 |
| *Both* | 1.14 | 0.875927 |
|  |  |  |
| **Drug availability** | |  |
| Essential obstetric drugs | 1.25 | 0.803036 |
|  |  |  |
| **Equipment** | |  |
| Sims speculum | |  |
| *No* | 1.19 | 0.837949 |
| Labour table | |  |
| *Available but unusable* | 1.15 | 0.867861 |
| *Not available* | 1.41 | 0.711374 |
| Bed Screen | |  |
| *Available but unusable* | 1.15 | 0.868845 |
| *Not available* | 1.29 | 0.775035 |
|  |  |  |
| **Infrastructure** | |  |
| Electricity | |  |
| *Irregular supply* | 1.88 | 0.530884 |
| *No connection* | 2.28 | 0.43885 |
| Water supply | |  |
| *Yes* | 1.14 | 0.87754 |
| Toilet |  |  |
| *Yes* | 1.24 | 0.80718 |
| Telephone | |  |
| *Yes* | 1.25 | 0.798962 |
|  |  |  |
| **Quality variables** | |  |
| SBA training in last 5 years | | |
| Y*es* | 1.1 | 0.908481 |
| VHSC monitoring work | | |
| *Yes* | 1.24 | 0.8044 |
|  |  |  |
| **Other variables** | |  |
| Region |  |  |
| *Central* | 4.85 | 0.205996 |
| *North-East* | 1.93 | 0.518195 |
| *East* | 2.5 | 0.399206 |
| *West* | 2.07 | 0.482912 |
| *South* | 2.77 | 0.361304 |
| ANM's residence from SC (in km) | | |
| *5-20* | 1.16 | 0.86154 |
| *21-40* | 1.07 | 0.935465 |
| *>40* | 1.02 | 0.980026 |
| Log of catchment population | 1.19 | 0.840834 |
| % population in lowest wealth quintile | 1.22 | 0.816472 |
| Total fertility rate | 2.56 | 0.391221 |
| % Hindu population | 1.52 | 0.659301 |
| Maternal education (in years) | 2.47 | 0.404214 |
| **Mean VIF** | **1.63** |  |
