# Supplementary material for: Supply-side barriers to maternal health care utilization at health sub-centers in India
Source: PeerJ. 2016 Nov 3;4:e2675. doi: 10.7717/peerj.2675 (PMC5101621; doi:10.7717/peerj.2675)
Supplement: Table S3 [file peerj-04-2675-s003.docx]

**Table A3: Variance inflation factors for postnatal care utilization model**

| **Variables** | **VIF** | **1/VIF** |
| --- | --- | --- |
| **Health personnel** |  |  |
| Auxiliary Nurse Midwife (ANM) |  |  |
| *None* | 1.16 | 0.864658 |
| *Only Contractual ANM* | 1.14 | 0.879539 |
| *Both* | 1.13 | 0.888326 |
|  |  |  |
| **Drug availability** | |  |
| Essential obstetric drugs | 1.26 | 0.792 |
|  |  |  |
| **Equipment** | |  |
| BP instrument | |  |
| *Yes* | 1.39 | 0.721 |
| Weighing scale | |  |
| *Yes* | 1.35 | 0.739 |
| Examination table | |  |
| *Available but unusable* | 1.09 | 0.920 |
| *Not available* | 1.29 | 0.774 |
| Bed Screen | |  |
| *Available but unusable* | 1.16 | 0.863 |
| *Not available* | 1.31 | 0.761 |
|  |  |  |
| **Infrastructure** | |  |
| Electricity | |  |
| *Irregular supply* | 1.90 | 0.526 |
| *No connection* | 2.30 | 0.435 |
| Water supply | |  |
| *Yes* | 1.14 | 0.880 |
| Toilet |  |  |
| *Yes* | 1.23 | 0.811 |
| Telephone | |  |
| *Yes* | 1.23 | 0.812 |
|  |  |  |
| **Quality variables** | |  |
| ISD training in last 5 years | | |
| *Yes* | 1.17 | 0.853 |
| SBA training in last 5 years | | |
| *Yes* | 1.15 | 0.869 |
| VHSC monitoring work | | |
| *Yes* | 1.24 | 0.808 |
|  |  |  |
| **Other variables** | |  |
| Region |  |  |
| *Central* | 4.92 | 0.203 |
| *Northeast* | 1.82 | 0.548 |
| *East* | 2.56 | 0.390 |
| *West* | 2.14 | 0.466 |
| *South* | 2.78 | 0.360 |
| ANM's residence from SC (in km) | | |
| *5-20* | 1.37 | 0.730 |
| *21-40* | 1.27 | 0.789 |
| *>40* | 1.07 | 0.933 |
|  |  |  |
| **Socioeconomic variables** |  |  |
| Log of catchment population | 1.19 | 0.843 |
| % population in lowest wealth quintile | 1.22 | 0.817 |
| Total fertility rate | 2.61 | 0.382 |
| % Hindu population | 1.56 | 0.641 |
| Maternal education (in years) | 2.50 | 0.399 |
| **Mean VIF** | **1.63** |  |
