# Supplementary material for: Supply-side barriers to maternal health care utilization at health sub-centers in India
Source: PeerJ. 2016 Nov 3;4:e2675. doi: 10.7717/peerj.2675 (PMC5101621; doi:10.7717/peerj.2675)
Supplement: Table S5 [file peerj-04-2675-s005.docx]

**Table A5: Results of the Park test for heteroscedasticity (delivery care model)**

| Test for heteroscedasticity | Result of test |
| --- | --- |
| Park Test | $\mu_{i}^{2}$= 0.476 + 0.543 *(estimated deliveries)  [70.40] [17.19]  R^2^ = 0.0181 |

$\mu_{i}$*represents the deviance residuals.*

*Values in* [] *represent the t-values associated with constant and β coefficient just above them.*

*Significant t-values of the explanatory variable suggest the presence of heteroscedasticity.*
