# Supplementary material for: Supply-side barriers to maternal health care utilization at health sub-centers in India
Source: PeerJ. 2016 Nov 3;4:e2675. doi: 10.7717/peerj.2675 (PMC5101621; doi:10.7717/peerj.2675)
Supplement: Table S6 [file peerj-04-2675-s006.docx]

**Table A6: Results of the Park test for heteroscedasticity (postnatal care model)**

| Test for heteroscedasticity | Result of test |
| --- | --- |
| Park Test | $\mu_{i}^{2}$= 1.149 – 0.0065 *(estimated postnatal care services)  [30.74] [1.42]  R^2^ = 0.0001 |

$\mu_{i}$*represents the deviance residuals.*

*Values in* [] *represent the t-values associated with constant and β coefficient just above them.*

*Significant t-values of the explanatory variable suggest the presence of heteroscedasticity*
