# Supplementary figures and images for: Supply-side barriers to maternal health care utilization at health sub-centers in India
Source: PeerJ. 2016 Nov 3;4:e2675. doi: 10.7717/peerj.2675 (PMC5101621; doi:10.7717/peerj.2675)

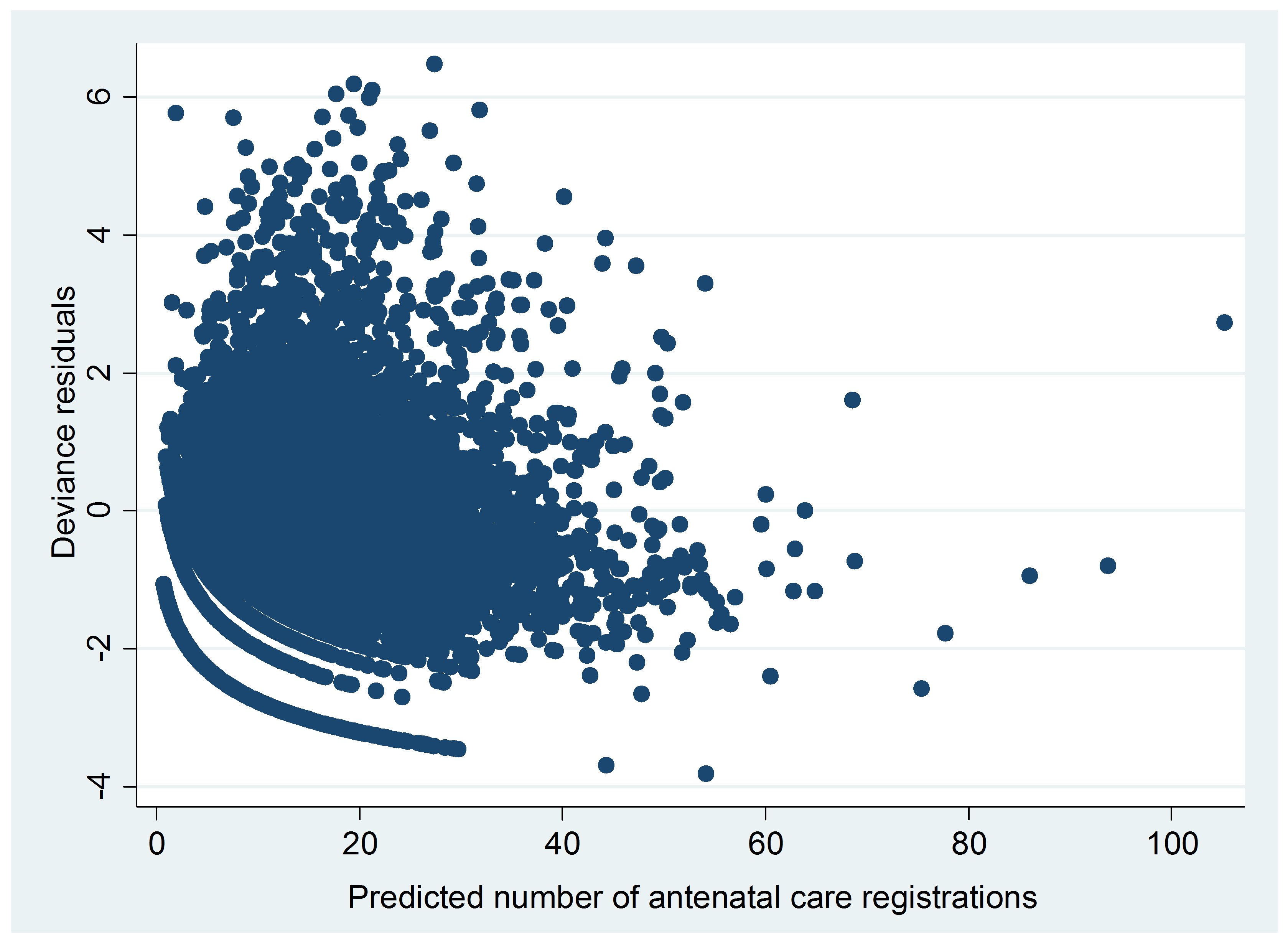

Supplement: Figure S1 [file peerj-04-2675-s007.png]

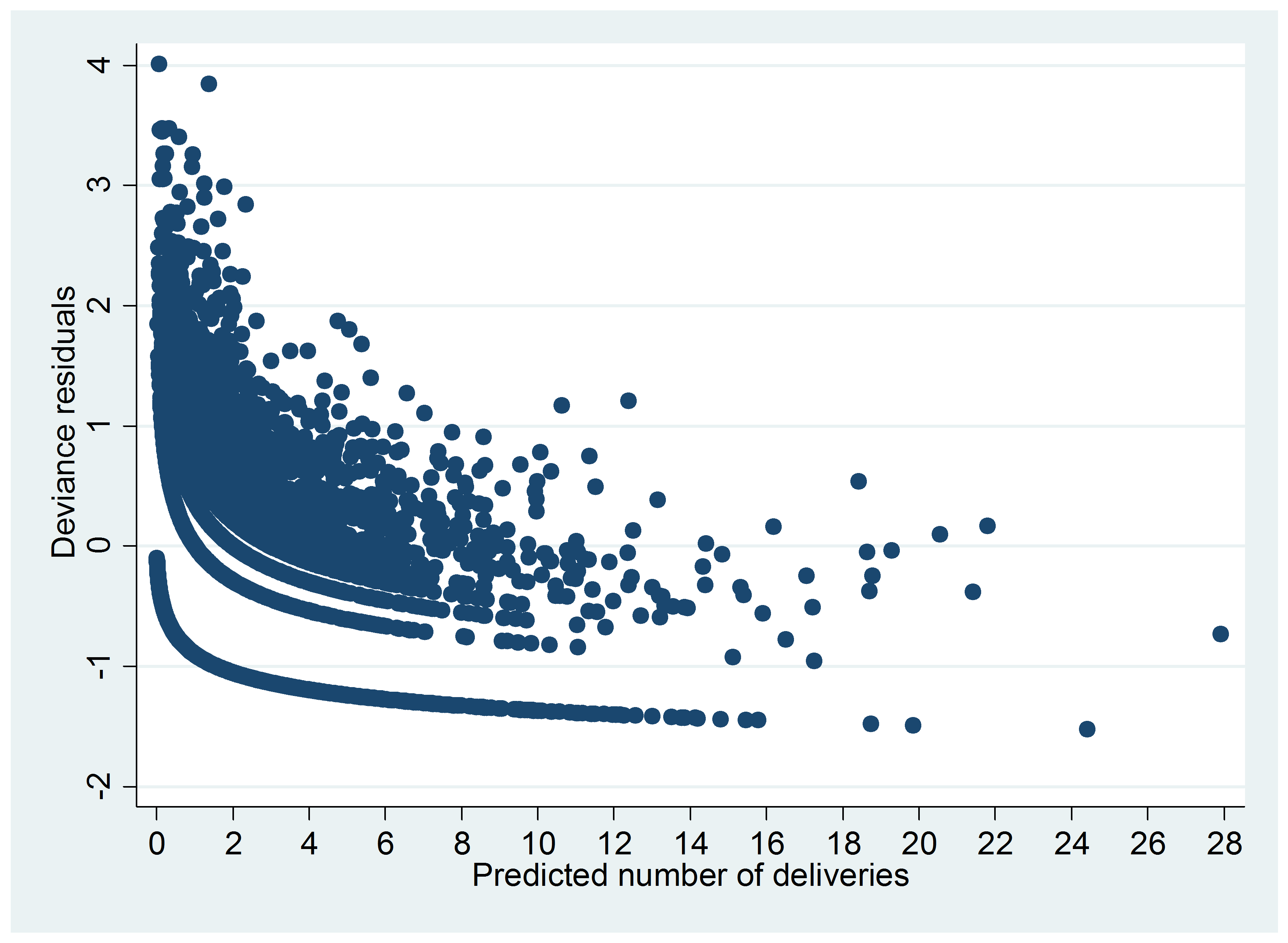

Supplement: Figure S2 [file peerj-04-2675-s008.png]

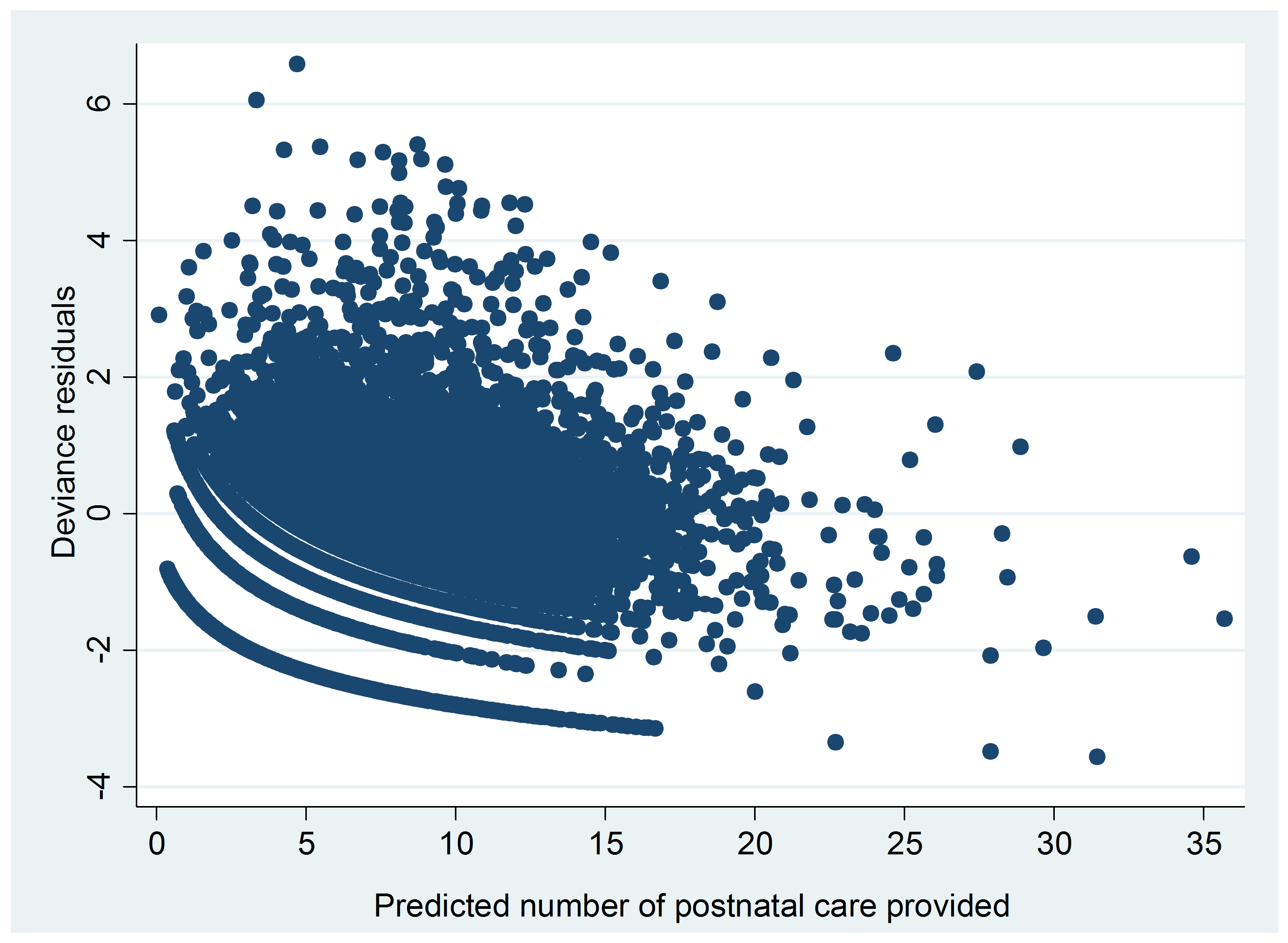

Supplement: Figure S3 [file peerj-04-2675-s009.png]
